# Predicting overall survival from tumor dynamics metrics using parametric statistical and machine learning models: application to patients with *RET*-altered solid tumors

**DOI:** 10.3389/frai.2024.1412865

**Published:** 2024-06-11

**Authors:** Erick Velasquez, Nastya Kassir, Sravanthi Cheeti, Denison Kuruvilla, Rucha Sane, Steve Dang, Dale Miles, James Lu

**Affiliations:** Clinical Pharmacology, Genentech Inc., South San Francisco, CA, United States

**Keywords:** oncology, survival prediction, pharmacometrics, statistical modeling, machine learning

## Abstract

In oncology drug development, tumor dynamics modeling is widely applied to predict patients' overall survival (OS) via parametric models. However, the current modeling paradigm, which assumes a disease-specific link between tumor dynamics and survival, has its limitations. This is particularly evident in drug development scenarios where the clinical trial under consideration contains patients with tumor types for which there is little to no prior institutional data. In this work, we propose the use of a pan-indication solid tumor machine learning (ML) approach whereby all three tumor metrics (tumor shrinkage rate, tumor regrowth rate and time to tumor growth) are simultaneously used to predict patients' OS in a tumor type independent manner. We demonstrate the utility of this approach in a clinical trial of cancer patients treated with the tyrosine kinase inhibitor, pralsetinib. We compared the parametric and ML models and the results showed that the proposed ML approach is able to adequately predict patient OS across *RET*-altered solid tumors, including non-small cell lung cancer, medullary thyroid cancer as well as other solid tumors. While the findings of this study are promising, further research is needed for evaluating the generalizability of the ML model to other solid tumor types.

## 1 Introduction

The modeling of tumor dynamics data has become widely utilized in supporting oncology drug development (e.g., see Bruno et al., 2020 for a recent review). Briefly, by quantitatively characterizing the temporal changes in tumor size under drug treatments, tumor growth inhibition (TGI) modeling generates metrics, which have shown to be predictive of patients' overall survival (OS) (Chan et al., 2021). Tumor size is typically represented by the sum of longest diameters (SLD) of, at most, five target lesions as outlined by RECIST 1.1 guidelines (Eisenhauer et al., 2009). Based on size measurements, TGI modeling generates the following on-treatment metrics: tumor regrowth rate (KG), tumor shrinkage rate (KS) and time to tumor regrowth (TTG) (Bruno et al., 2023). KG refers to the rate at which the tumor regrows while the patient is still undergoing treatment, where a lower KG value might suggest the treatment is effectively reducing the growth of the tumor. KS represents how quickly the size of the tumor is reducing under the effect of the treatment, where a higher KS value would typically represent a more effective treatment. TTG indicates the time it takes for the tumor to start growing again after start of treatment, where a longer TTG indicates that the treatment has a lasting effect on halting the tumor's growth. TGI modeling and the resulting metrics provide valuable insights into the potential effectiveness of a therapy and be predictive of patients' OS (Chan et al., 2021). In much of the existing modeling efforts, the relationships between TGI metrics and OS are identified in an indication-specific manner (e.g., in the reference Chan et al., 2021), whereby the assumption is made that this quantitative link is drug-independent but disease-specific. Although the current approach demonstrably assists decision-making in frequently encountered cancer indications with significant historical patient data (Bruno et al., 2023), its application may be limited in clinical trials involving treatment arms for rarer tumor types or those that are tumor type-agnostic.

The challenge of predicting patient survival in a tumor type agnostic manner calls for a machine learning (ML) framework (Duda et al., 2021). In this work, we applied a ML model that accounts for patient baseline covariates as well as TGI metrics to predict OS. Specifically, the training set consists of comprehensive patient data collected across 10 clinical trials representing five tumor types. For our survival model, we leveraged the XGBoost algorithm (Chen and Guestrin, 2016), following successful implementation in a prior study (Laurie and Lu, 2023).

In addition to making accurate and generalizable predictions, ML models also need to be explainable in order to ensure human involvement in the final decision-making process (Terranova et al., 2023). Toward this goal, the methodology of Shapley additive explanation (SHAP) was introduced (Lundberg et al., 2020; Terranova et al., 2023) and has been previously applied to explain ML models of patient survival (Sundrani and Lu, 2021; Laurie and Lu, 2023).

In this work, we compare the parametric statistical and ML models for OS predictions, in a setting involving patients with solid tumors across several tumor types—the ARROW study. The trial stands as a robust case study for methodological comparison, given its expansive scope examining oncogenic *RET* alterations across a diverse range of solid tumors, like Non-Small Cell Lung Cancer (NSCLC) and Medullary Thyroid Cancer (MTC), and other solid tumors.

## 2 Methods

### 2.1 Clinical study data

The ARROW study (NCT03037385, BO42863) is a multi-center, open-label, phase I/II study evaluating the safety, efficacy, and pharmacokinetics of pralsetinib in patients with advanced solid tumors associated with oncogenic alterations in the rearranged during transfection (*RET*) gene (Claret et al., 2013; Subbiah et al., 2021), which is a key player in certain types of cancers. Alterations in the *RET* proto-oncogene, which encodes a transmembrane receptor tyrosine kinase, has been implicated in molecular pathogenesis of many solid tumors (Subbiah et al., 2021). Tumor response, as a measurement of SLD of target lesions per RECIST 1.1 guidelines (Eisenhauer et al., 2009), was assessed in patients with RET fusion-positive solid tumors (Claret et al., 2013; Subbiah et al., 2021). The trial has been previously described (Gainor et al., 2021; Subbiah et al., 2021) and spans oncogenic *RET* alterations in NSCLC (*n* = 313), MTC (*n* = 224) and other advanced solid tumors exclusively with a *RET* fusion (OTHER FUS, *n* = 28) or any other *RET* alteration or rearrangement (OTHER ALT, *n* = 20) (data cutoff date: March 4, 2022).

### 2.2 Generation of TGI metrics

The TGI model was constructed as outlined in previous work (Stein et al., 2008). Briefly, a biexponential TGI model (Claret et al., 2018) was fit to longitudinal tumor size data from the ARROW study. The model was executed as a nonlinear mixed effect model using NONMEM version 7.5. Patients with baseline and at least one other post-baseline tumor lesion measurement were considered TGI-evaluable (Rittmeyer et al., 2017) and included in the parametric analysis (*n* = 556, consisting of: 294 in NSCLC, 217 in MTC, 27 in OTHER FUS, 18 in OTHER ALT); ML model was able to leverage all patients, including TGI non-evaluable patients. Variability in KG and KS related to tumor type was characterized under the assumption of a log-normal distribution, and an additive residual error was described using a normal distribution. The performance of the model was assessed using standard goodness-of-fit plots. Notably, TGI metrics derived using this model were utilized in both the parametric TGI-OS model as well as the pan-indication ML model.

### 2.3 Parametric TGI-OS model

A parametric statistical TGI-OS model was developed (Stein et al., 2008) utilizing data compiled from six atezolizumab NSCLC clinical trials (3,872 patients) as previously described (Fehrenbacher et al., 2016; Reck et al., 2019; West et al., 2019; Jotte et al., 2020; Nishio et al., 2021). The influence of potential covariates present in the training dataset, with explanatory variables consisting of TGI metrics and baseline prognostic factors, on OS were examined via the Kaplan–Meier method and subsequently analyzed through univariate screening using Cox regression analysis. The complete set of parameters for the model were determined using parametric survival regression, where the most suitable probability density function that accurately represented the observed data was chosen. A backward stepwise elimination process was then performed, considering a significance threshold of *p-*value < 0.01, and retaining only the covariates which were significant. The evaluation of model development was conducted using the R programming language version 4.1.1.

Through this procedure, covariates which were found to be significant (with a *p-*value < 0.05) included: TGI metric log (KG), inflammatory markers such as baseline albumin and the neutrophil to lymphocyte ratio (NLR), along with common prognostic factors such as baseline Eastern Cooperative Oncology Group performance status (ECOG), baseline sum of longest diameter, presence of liver metastasis, and number of tumor sites. Furthermore, patient race (Asian vs. non-Asian) and gender were also identified as significant covariates.

### 2.4 Pan-indication ML model for TGI-OS

The pan-indication ML model utilizes a comprehensive dataset comprising of 8,121 patients, spanning across five distinct types of cancer (NSCLC, small cell lung cancer, renal, triple-negative breast cancer, urethral) from all arms in the 10 atezolizumab containing clinical trials, with drug treatments covering both small and large molecules as have been previously described (Fehrenbacher et al., 2016; Horn et al., 2018; Powles et al., 2018; Reck et al., 2019; Rini et al., 2019; West et al., 2019; Schmid et al., 2020; Chan et al., 2021; Nishio et al., 2021; Vieira et al., 2021). The input features incorporate all three TGI metrics (i.e., KS, KG and TTG) as well as 8 baseline covariates [ECOG, Hemoglobin (HGB), Albumin (ALBU), NLR, liver metastasis status, number of tumor sites, neutrophil count (NEU), and number of years since initial diagnosis (YSD)]. Notably, the ML model is tumor type agnostic, as the tumor type is not an input variable.

We used XGBSE (Vieira et al., 2021), a gradient boosting framework with survival embeddings, with hyperparameters set as summarized in the Supplementary material. XGBSE model was then fitted to training data (TGI metrics and baseline covariates across 10 atezolizumab containing trials described above) and predictions were made calculating the probability of an event from 0 to 2,000 days, in time intervals of 5 days. Bootstraps were performed to generate 1,000 model predictions, from which 95% prediction intervals were generated (see Laurie and Lu, 2023 for further details). Additionally, SHAP values (Lundberg et al., 2020) were calculated on the trained model by using the TreeExplainer.

## 3 Results

### 3.1 OS and TGI metrics differ between indications

**Figure 1:**
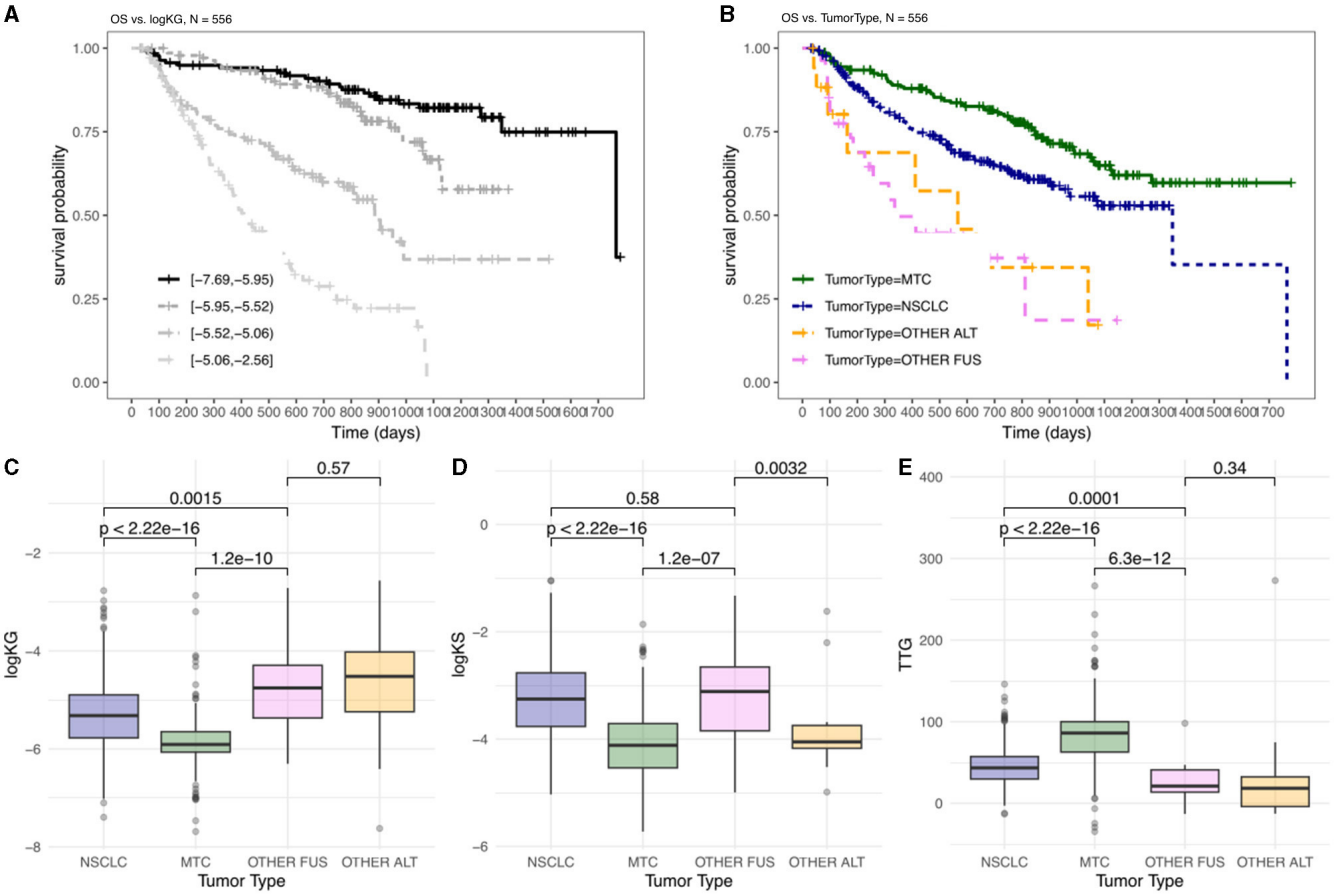
OS and TGI metrics differ between indications amongst patients treated with pralsetinib. Kaplan–Meier plots are shown, stratified by: **(A)** four equal groups of patients stratified by calculated log(KG) values; **(B)** tumor type. Distribution of TGI metrics, logKG **(C)**, logKS **(D)**, and TTG **(E)**, by tumor type, with pairwise comparison summarizing significant differences by p-values. OTHER FUS, other RET fusion; OTHER ALT, other RET alterations.

A key aim of this study is to assess the applicability of Tumor Growth Inhibition (TGI) Overall Survival (OS) modeling across various tumor types, in a tumor agnostic setting. To begin to understand its applicability, we first studied how TGI metrics amongst TGI-evaluable patients in the pralsetinib trial varied across different tumor types and analyzed their relationships with OS (Figure 1).

Log(KG) has been previously shown to be the most important predictor of OS in TGI-OS modeling across several solid tumors (Chan et al., 2021). When stratifying patients by log(KG) there was a difference in OS whereby patients with lower log(KG) (i.e., smaller KG value) showed longer OS compared to patients with higher log(KG) (Figure 1A). When the data was examined in relation to tumor type, a significant variation in overall survival was observed across different tumor types. Notably, patients with MTC exhibited the best OS (Figure 1B). This could indicate that patients with distinct tumor types might respond differently to the therapy. To discern whether variation between indications - potentially reflecting both disease differences and differential drug effects - were encapsulated in the tumor dynamics, we examined TGI metrics across these indications (Figures 1C–E).

When considering log(KG), there is a significant difference between MTC, NSCLC and Other *RET* fusion-positive (FUS)/*RET*-altered (ALT) (i.e., excluding *RET* fusion-positive) tumors (Figure 1C). These differences align with the observed OS, since patients with smaller KG (Figure 1C, MTC) would show less tumor growth and thus have a better prognosis (Figure 1B, MTC) compared to patients with higher tumor growth rates (Figures 1B, C, OTHER FUS/ALT). When considering TTG, there is a significant difference between MTC, NSCLC and Other FUS/ALT tumors (Figure 1E). These differences align with the observed overall survival, since patients with longer TTG (Figure 1E, MTC) would have a better prognosis (Figure 1B, MTC) compared to patients with shorter TTG (Figure 1, OTHER FUS/ALT).

### 3.2 ML model enables reliable patient survival predictions across tumor types

**Figure 2:**
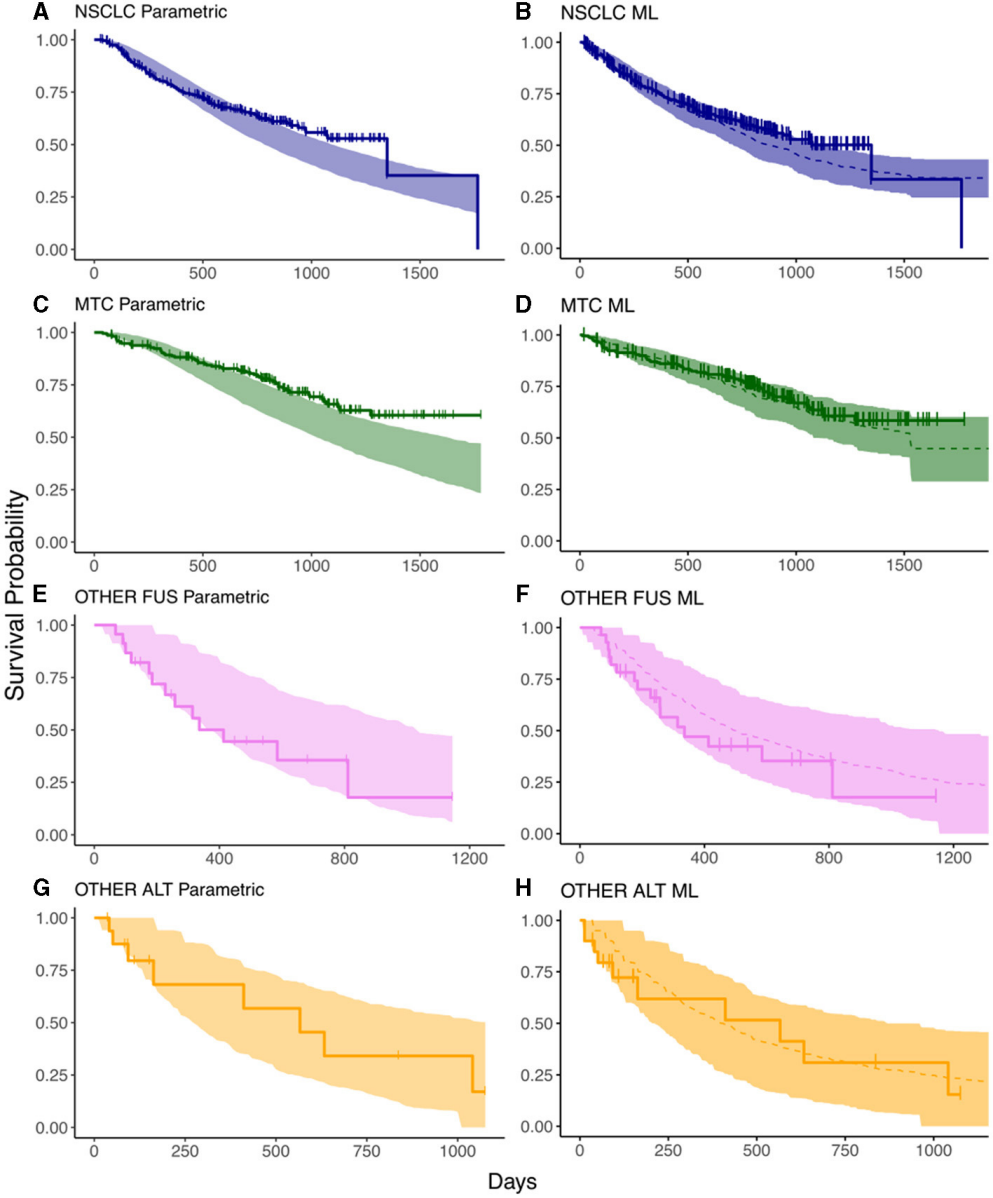
Prediction intervals from survival models compared against the data from the ARROW trial. **(A, C, E, G)** are the results from the parametric survival model in each of the tumor types; **(B, D, F, H)** are those from the ML model. Solid lines represent the observed KM curves while shaded regions represent prediction intervals. The dashed lines indicate the median predicted survival in the ML model. The horizontal axis indicates time in days, the vertical axis indicates survival probability.

The OS prediction results from the parametric and ML models are shown in Figure 2, where the shaded bands indicate the 95% prediction intervals (PIs) and the solid lines with marked crosses are the Kaplan–Meier curves of the observed survival data. The results indicate that the ML predictions exhibit superior accuracy across the array of tumor types: in patients with NSCLC, there is a fair agreement between the parametric and ML model in predicting OS (Figures 2A, B); however, there is a discrepancy in predictions amongst patients with MTC, with the parametric modeling predicting a worse survival than what is observed at the end of the survival interval (Figure 2C). The TGI-OS ML method captures this difference, predicting an OS that aligns with the observed data across the survival interval (Figure 2D). Predictions for patients with other *RET* fusion-positive/*RET*-altered cancers were similar between the parametric and ML methods and is characterized by wider prediction intervals, partly reflecting the small number of patients in these groups (Figures 2E–H).

### 3.3 Explaining the input dependence in the ML model

**Figure 3:**
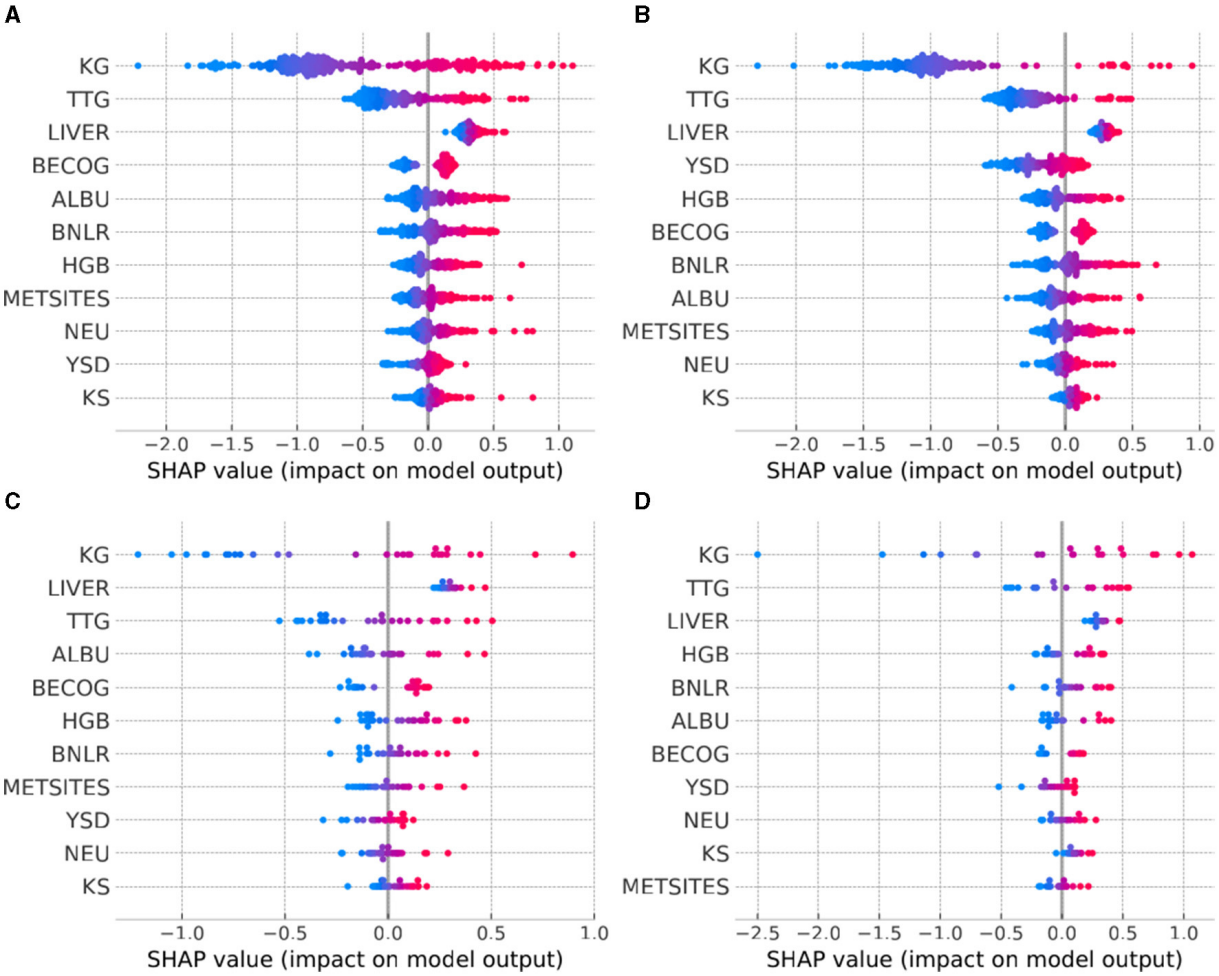
Model explanation provided by SHAP summary plots across the tumor types of interest. **(A)** NSCLC, non-small cell lung cancer; **(B)** MTC, medullary thyroid cancer; **(C)** OTHER FUS, other RET fusion-positive; **(D)** OTHER ALT, other RET-altered. For each tumor type, the explanatory variables are ordered from top to bottom according to decreasing level of influence. The color of the points represents the value of the explanatory variables: red indicates high and blue indicates low values.

In our study, we utilized Shapley Additive exPlanations (SHAP) (Lundberg et al., 2020) to understand each feature's contribution to the hazard prediction in patients. The most important feature contributing to the hazard rate, as observed across various tumor types, is KG (Figures 3A–D), as visually indicated by the broad dispersion of the SHAP values from the zero value. The SHAP values for KG also aligned with what would be expected for hazard; for example, patients with high KG values (Figures 3A–D: red points) exhibited high SHAP values, affirming that having a high KG contributes to predicting higher hazard. Interestingly, another TGI metric—TTG, also demonstrated notable importance across distinct tumor types. The fact that two main TGI metrics (KG, TTG) exhibited high importance is in agreement with existing parametric TGI modeling work showing these parameters as the most predictive of hazard rates. However, KS showed consistently lower importance across different tumor types (Figures 3A–D), suggesting that the initial tumor remission does not have a strong influence on the hazard rate beyond those provided by KG and TTG. Finally, the importance of remaining features was not consistent across all tumor types. This result emphasizes the patient variation across indications and their contribution to hazard.

## 4 Discussion

While the methodology of using TGI metrics to predict OS is well established in supporting oncology drug development, it becomes challenging when the clinical trial to be predicted involves tumor types not present in the available historical dataset. In this study, we examined how this obstacle could be overcome by utilizing an ML model that has been trained on a large number of patients across distinct solid tumor tumor types. We showed that whereas using a parametric TGI-OS model that has been fitted to NSCLC may lose predictivity when applied to a different tumor type (e.g., MTC), the pan-tumor ML model is able to adequately describe the OS curves across both NSCLC and MTC, as well as for subsets of patients who have a variety of tumor types. While it is feasible to attempt a parametric TGI-OS model using the same dataset and explanatory variables as the ML model, its effectiveness in modeling a heterogeneous dataset is unproven and becomes significantly challenging to scale with the increase in data volume. The results indicate the potential capability of the pan-indication ML model in extrapolating to new solid tumor types that are not in the training set, which opens the door for increasing the future applications of the TGI-OS modeling approach. This is especially evident in the OS predictions for MTC, where the ML model better predicts OS toward the end of the survival curve. We believe that having a diverse and more heterogeneous training set provides the ML model with more flexibility when making OS predictions compared to parametric statistical models, by relaxing assumptions such as proportional hazards and linearity between explanatory variables and hazard rates. Coupled with the explainability technique of SHAP analysis, the pan-indication model can further elucidate similarities as well as differences between indications and hence help support future clinical studies.

While the results reported in this study are encouraging, there are some potential limitations in the prospective use of this methodology. Firstly, the current models take as input the tumor metrics, which require sufficient clinical follow-up in order to obtain accurate estimates. Deep learning could provide a solution to this limitation, allowing for the generation of tumor metrics from shorter follow-ups, a process explored in Laurie and Lu (2023). Furthermore, the generalizability of the ML model to additional solid tumor types remains to be further validated in future research.

## Data availability statement

The data analyzed in this study is subject to the following licenses/restrictions: Qualified researchers may request access to individual patient level data through the clinical study data request platform (https://vivli.org/). Further details on Roche's criteria for eligible studies are available here (https://vivli.org/members/ourmembers/). For further details on Roche's Global Policy on the Sharing of Clinical Information and how to request access to related clinical study documents, see here (https://www.roche.com/research_and_development/who_we_are_how_we_work/clinical_trials/our_commitment_to_data_sharing.htm). Requests to access these datasets should be directed to https://vivli.org/.

## Ethics statement

The studies involving humans were approved by Institutional Review Boards at all study sites. The studies were conducted in accordance with the local legislation and institutional requirements. The participants provided their written informed consent to participate in this study.

## Author contributions

EV: Formal analysis, Investigation, Writing—original draft, Writing—review & editing. NK: Conceptualization, Formal analysis, Methodology, Supervision, Validation, Writing—original draft, Writing—review & editing. SC: Investigation, Project administration, Writing—original draft, Writing—review & editing. DK: Data curation, Writing—original draft, Writing—review & editing. RS: Investigation, Project administration, Writing—original draft, Writing—review & editing. SD: Data curation, Writing—review & editing. DM: Investigation, Project administration, Writing—original draft, Writing—review & editing. JL: Conceptualization, Methodology, Supervision, Validation, Writing—original draft, Writing—review & editing.
